# 

**Published:** 1906-06-02

**Authors:** 


CONTENTS.

**Alcohol in Relation to Therapeutics** - - - - 149

**The Visit of Metchnikoff** - - - - - - - - 150

**Annotations** - - - - - - - - - - - 151

**Medical Opinion and Movement** - - - - - - 152

**Hospital Clinics—**

The Present Position of Radiation in Treatment - - - - 153

On Typhoid Fever - - - - : - - - - - - 155

Broncho pneumonia and Abscess of the Lung - - - - - 157

**Progress in Medicine and Surgery—**

Nervous Diseases - - - - - - - - - - 158

**New Appliances and Things Medical** - - - - - 159

**The Book World of Medicine and Science** - - 160

**Medical Appointments and Vacancies** - - - 160

**Hospital Administration—**

Current Hospital Topics - - - - - - - - - - 161

British Red Cross Society - - - - - - - - - 162

Surgical Homes - - - - - - - - - - - - 164

The American Ambassador on Hospitals - - - - - - 164

King's College Hospital - - - - - - - - - - 165

Social and Poor-law Problems - - - - - - - - 166

Hospital Meetings - - - - - - - - - - - - 167

Help the Defectives . - - - - - - - - - - 170

**Notes and News** - - - - - - - - - - - 170

*NURSING SECTION.*

**Notes on News from the Nursing World—**
Meeting at the Colonial Office—Asylum Workers and Pensions—Night-Nursing in Poor-law Infirmaries—Army Nursing in America—Nursing Europeans in India—Answering Questions in Writing—District Nursing Fees—The Late Miss Alice Phillimore—King's College Hospital Fête—Mr. Pawling's Home—School Nurses—A Memorial to a Pioneer—Proposed Surgical Hostel for Gentlewomen—Devonshire Imitating Plaistow—Splendid Bequest to a Nursing Institute—Brentford Union Infirmary—Short Items - - - - - - 127-130
**The Nursing Outlook** - - - - - - - - - 131
**The Care and Nursing of the Insane** - - - - - 132
**The Nurse's Clinic** - - - - - - - - - - 133
**Incidents in a Nurse's Life** - - - - - - - - 134
**The Jubilee of the London Biblewomen and Nurses Mission** - - - - - - - - - - 135
**Central Midwives Board** - - - - - - - - - 136
**Canon Scott-Holland on Nurses** - - - - - - 137
**Nurses' Missionary League** - - - - - - - - 137
**Everybody's Opinion** - - - - - - - - - - 137
**Appointments** - - - - - - - - - - - - 138
**Presentations** - - - - - - - - - - - - 138
**Death in Our Ranks** - - - - - - - - - - 139
**Novelties for Nurses** - - - - - - - - - 139
**Notes and Queries** - - - - - - - - - - 140
**For Reading to the Sick** - - - - - - - - 140

